# High sensitivity C-reactive protein levels in Acute Ischemic Stroke and subtypes: A study from a tertiary care center

**Published:** 2013

**Authors:** Jaydip Ray Chaudhuri, Kandadai Rukmini Mridula, Matapathi Umamahesh, Alluri Swathi, Banda Balaraju, Venkata Chandrasekher Srinivasarao Bandaru

**Affiliations:** 1 Department of Neurology, Yashoda Hospital, Hyderabad, India; 2 Assistant Professor, Department of Neurology, Nizams's Institute of Medical Sciences, Hyderabad, India; 3 Department of Radiology, Yashoda Hospital, Hyderabad, India; 4 Clinical Register, Department of Neurology, Yashoda Hospital, Hyderabad, India; 5 Department of Medicine, Yashoda Hospital, Hyderabad, India; 6 Research Coordinator, Department of Neurology and Clinical Research, Yashoda Hospital, Hyderabad, India

**Keywords:** HsCRP, Ischemic Stroke, Stroke Subtypes, Indian Patients

## Abstract

**Background:**

Stroke is a heterogeneous disease with several risk factors. High sensitivity C-reactive protein (hsCRP) is a marker for cardiovascular and cerebrovascular diseases. Recent studies have shown that high hsCRP level is a risk factor for ischemic stroke. The objective of our study was to investigate the association of high hsCRP (> 3 mg/L) levels with ischemic stroke and its subtypes in Indian patients.

**Methods:**

We recruited 210 consecutive acute stroke patients and 150 age and sex matched controls. Stroke patients were admitted within 72 hours of onset, at Yashoda Hospital, Hyderabad, India. The study period was from January 2011 to December 2012. All patients underwent tests as per standard protocol for stroke workup. Serum hsCRP level was assessed in all stroke patients and controls on the day of admission.

**Results:**

The mean hsCRP was significantly higher in stroke patients (3.8 ± 2.5) than controls (1.8 ± 1.5) (P < 0.001). High hsCRP had higher frequency in stroke patients 130 (61.9%) compared to controls 10 (6.6%), P < 0.001. High hsCRP level was more prevalent in the stroke subtypes of cardioembolic stroke (83.3%) and large artery atherosclerosis (72%). High hsCRP level was significantly associated with hypercholesterolemia (P = 0.001), age (P = 0.01), and mortality (0.04). After adjustment of regression analysis it was observed that high level hsCRP is independently associated with acute ischemic stroke (Odds 4.5; 95% CI: 2.5-12.2); especially the stroke subtypes of cardioembolic stroke, (odds ratio 3.4, 95% CI: 1.9-10.5) and large artery atherosclerosis (odds ratio 2.1, 95% CI: 1.5-3.8).

**Conclusion:**

High hsCRP level is strongly associated with and an independent predictor of acute ischemic stroke. The association was found in all ischemic stroke subtypes.

## Introduction

Infections and inflammation play a vital role in the pathophysiology of atherosclerosis.^1–3^ High sensitive C-reactive protein (hsCRP) is a sensitive marker of inflammation and tissue injury in the arterial wall.^4, 5^ CRP is a glycoprotein produced by the liver and plays a vital role in the development of atherosclerotic disease in cardiac and cerebral circulation.^6–10^

Cerebrovascular ischemia has been associated with bronchial and periodontal infections.^11^ As a marker of infection and inflammation, high hsCRP has been associated with acute stroke.^9, 12^ Since infectious and inflammatory diseases are more common in India compared to western countries, we aim to investigate the association of high hsCRP (> 3 mg/L) levels in patients with ischemic stroke and its subtypes. A very limited number of studies are available from India on the association of hsCRP with ischemic stroke subtypes.

## Materials and Methods

210 consecutive patients with acute ischemic stroke were included in the study and were enrolled from the Department of Neurology, Yashoda Hospital, Hyderabad, India. Stroke patients were enrolled into the study if they met the following criteria: first ischemic stroke, and admission to the hospital within 72 hours of onset of stroke. The exclusion criteria were transient ischemic attack, recurrent stroke or second stroke, intracerebral haemorrhage, subarachnoid haemorrhage, lack of baseline data, and admission to the hospital over 72 hours of the onset of stroke. Patients with clinical symptoms and signs of active infection including fever, cough, burning micturition, and asymptomatic subjects with evidence of infection on investigations such as leucocytosis on peripheral smear, pus cells in urine, infiltrates on chest radiograph, since they may cause elevation of hsCRP, were excluded. Patients with a history of prior inflammatory diseases, like rheumatoid arthritis and systemic lupus erythematosus (SLE), and those on steroids or immunomodulatory drugs were also excluded. Stroke was defined according to the World Health Organization as “rapidly developing clinical signs of focal/global disturbance of cerebral function, with symptoms lasting 24 hours or longer or leading to death, with no apparent cause other than of vascular origin”.^13^ Cerebral infarction was diagnosed on the basis of history, neurological examination, and neuroimaging (CT or brain MRI). All subtypes of ischemic stroke were included.

150 age and sex matched control subjects were recruited from the same hospital. Controls were healthy subjects chosen from patients with no present or past history of stroke, transient ischemic attack (TIA), or cardiac disease. Yashoda Hospital is a major referral centre in Andhra Pradesh state. The study period was two years, from January 2011 to December 2012. This study was approved by the Institutional Ethical Committee.

### Stroke subtypes assessment

All stroke patients underwent brain imaging by computerised tomography (CT) scan, and when clinically appropriate magnetic resonance imaging (MRI) and intracranial magnetic resonance angiography (MRA) of the brain. Cardiac evaluation with transthoracic echocardiography (TTE) or transesophageal echocardiography (TEE), and non-invasive vascular imaging (extracranial duplex Doppler) were done in all patients. Additional tests were performed when required. The stroke specialist reviewed the data and subclassified the strokes as extracranial large artery atherosclerosis, intracranial large artery atherosclerosis, cardioembolic, small vessel disease (lacunar), stroke of other determined etiology, and stroke of undetermined etiology.^14^

Standardized techniques were adapted from the behavioural risk factor surveillance system.^15^ Data were collected through face-to-face interviews of patients, and physical and neurological examination by a stroke specialist. When the subjects were unable to provide answers, their close relations who were knowledgeable about the subject's history were interviewed. All stroke patients and control subjects underwent blood tests which included fasting blood sugar, lipid profile, homocysteine levels, and other biochemical and haematological tests. Collagen diseases profile and tests for prothrombotic state were done, if stroke subtype was not clear. Risk factor definitions were the same as in our previous publication.^16^

### Estimation of hsCRP

Levels of hsCRP were estimated by VITROS 5.1 chemistry system and VITROS 5600 integrated system to quantitatively measure CRP in human serum or plasma. As per the normative data from VITROS 5600 system manual and current literature, the cardiovascular risk was determined as low risk with hsCRP levels < 1.0 mg/L, medium risk if 1.0-3.0 mg/L, high risk when > 3.0 mg/L.^12^ For our study we considered hsCRP level of ≥ 3 mg/L as high risk and ≤ 3 mg/L as low risk.^12^

### Statistical analysis

Statistical analysis was done using the Statistical Package for Social Sciences (SPSS 16.0, SPSS Production Facility, Chicago, Illinois, USA). Continuous variables were presented in titre of mean ± SD. Categorical variables were expressed as proportions, and chi-square test was used to study the association in proportions. All tests were two sided and p values < 0.05 were considered statistically significant. We performed multiple logistic regression analysis for stroke subtypes and high hsCRP.

## Results

We recruited 210 acute ischemic stroke patients and 150 age and sex matched control subjects for the period of two years. On comparison of hsCRP levels and other vascular risk factors among stroke patients and control subjects it was found that a significantly higher proportion of stroke patients had hypertension, diabetes, and high hsCRP levels (Table 1).

**Table 1:** Demographic parameters, vascular risk factors, and hsCRP levels among stroke patients and control subjects

Parameter	Stroke patients (n = 210) (%)	Control Subjects (n = 150)	P
Men	152 (72.3%)	108 (72%)	0.100
Mean age	61.2 ± 14.2	62.4 ± 15.9	0.200
Age range	23-87	25-87	
Mean hsCRP	3.8 ± 2.5	1.8 ± 1.5	< 0.001
high hsCRP	130 (61.9%)	10 (6.6%)	< 0.001
Hypertension	128 (60.9%)	36 (24%)	< 0.001
Diabetes	96 (45.7%)	32 (21%)	< 0.001
Smoking	89 (42.3%)	50 (33.3%)	0.100
Alcoholism	86 (40.9%)	56 (37.3%)	0.300
Hypercholesterolemia	82 (39%)	35 (23%)	0.300
Hyperhomocysteinemia	25 (11.9%)	15 (10%)	0.400
Deaths	9 (4.2%)	0	0.010

The prevalence of high hsCRP level was highest in patients with cardioembolic stroke 83.3% (20/24), followed by large artery atherosclerosis 72% (62/86) (Table 2).

**Table 2:** High hsCRP associated with stroke subtypes

Stroke subtypes	Number (130/210)
Large artery atherosclerosis	62/86 (72%)
Cardioembolic stroke	20/24 (83.3%)
Small artery disease	17/34 (50%)
Stroke of other determined etiology	6/15 (40%)
Stroke of undetermined etiology	25/51 (49%)

Out of 210 stroke patients, high hsCRP levels were detected in 130 (61.9%). On comparison between high and low hsCRP groups it was found that hypercholesterolemia, older age, and mortality were significantly associated with high hsCRP levels (Table 3).

**Table 3:** Comparison between high hsCRP (> 3.0mg/L) and average hsCRP (≤ 3.0mg/L) with ischemic stroke patients

Parameters	High hsCRP (n = 130) (> 3.0mg/L)	Low hsCRP (n = 80) (≤ 3.0mg/L)	P
Men	95 (73%)	38 (47.5%)	0.003
Mean age	64.9 ± 14.3	59.8 ± 13.5	0.010
Risk factors			
Hypertension	83 (63.8%)	45 (56.2%)	0.270
Diabetes	53 (40.7%)	43 (53.7%)	0.090
Smoking	42 (32.2%)	29 (36.2%)	0.600
Alcoholism	53 (40.7%)	33 (41.2%)	0.900
Hypercholesterolemia	60 (46.1%)	22 (27.5%)	0.001
Hyperhomocysteinemia	14 (10.7%)	8 (10%)	0.900
Death	7 (5.3%)	2 (2.5%)	0.040

After adjustment of multiple logistic regression analysis, high hsCRP was independently associated with acute ischemic stroke (OR 4.5; 95% CI 2.5–12.2) overall, and in stroke subtypes of cardioembolic stroke (OR 3.4; 95% CI 1.1–10.5) and large artery atherosclerosis (OR 2.1; 95% CI 1.1–3.8) (Table 4).

**Table 4:** Before and after adjusted odds ratio analysis between presence of high hsCRP in stroke subtypes (in stepwise method)

Stroke subtypes	Before adjusted odd ratio	After adjusted odd ratio

	Odds ratio (95% CI)	Odds ratio(95% CI)
All stroke subtypes combined	8.2 (5.4-15.5)	4.5 (2.5-12.2)
Large artery atherosclerosis	2.7 (1.9-5.1)	2.1 (1.5-3.8)
Cardioembolic stroke	5.4 (2.7-16.9)	3.4 (1.9-10.5)
Small artery disease	0.5 (0.2-1.1)	0.2 ( 0.1-0.4)
Stroke of other determined etiology	0.3 (0.1-1.1)	*
Stroke of undetermined etiology	0.6 (0.2-0.9)	0.3 (0.1-0.6)

* Number of patients insufficient for statistical analysis

## Discussion

In this present prospective study, more than three fifths of Indian patients with acute ischemic stroke had high hsCRP (> 3 mg/l) levels. Other studies have shown varying prevalence. Rajput et al. had found that among stroke patients from Pakistan, 132 (88%) had elevated CRP (CRP > 10 mg/L).^17^ Moreover, in a study by Di Napoli et al. from Italy, 95 patients (74.2%) with acute ischemic stroke had high CRP levels (> 0.5 mg/dl) at admission.^18^ Muir et al. had detected elevated CRP (> 10 mg/L) levels in 96 out of the228 (42.1%) patients admitted with acute ischemic stroke in the UK.^19^ On the other hand, only 22% of stroke patients and 14% of myocardial infarction patients had high CRP (> 7 mg/l) levels in a study from Netherlands.^20^ This variance may be explained partly by the different definitions of high CRP in various studies. The hsCRP levels are now becoming universally standardised and most centres accept a value above 3 mg/dl as high.^12^

CRP has evolved from being an association to a risk factor for vascular pathology of heart and brain. Zacho et al., in his population based study, found a high frequency of ischemic heart disease (32%) and ischemic stroke (25%) among patients with high levels of CRP in Denmark.^21^ Ridker et al. from the US, showed high CRP to be a predictor of risk for future myocardial infarction and stroke in healthy men.^22^ Among the Japanese population Arima et al. showed a significant association between high hsCRP and future risk of coronary artery disease.^23^ Moreover, CRP has also been associated with poorer outcomes in cardio and cerebrovascular diseases. In recent studies evaluating various biomarkers, including atrial and brain natriuretic peptides, CRP, and homocysteine, in outcome of stable cardiovascular diseases showed that CRP was associated with an increased risk of congestive heart failure.^24^ Thus there is increasing evidence that hsCRP is a risk as well as prognostic factor for ischemic stroke and coronary events.^10, 20, 25, 26^

### Stroke subtypes

In this study, high hsCRP levels were associated with all stroke subtypes. Prevalence of high hsCRP was maximum in cardioembolic stroke (83.3%) followed by large artery atherosclerosis (both intracranial and extracranial) (72%) and small artery disease (50%).

### Large artery atherosclerosis

Among our patients with large artery atherosclerosis, high hsCRP levels were found in 72%. This is similar to reports by Huang et al. (63.9% in large artery atherosclerosis)^12^ and Rajeshwar et al.^27^ who observed high CRP in both intracranial (48.7%) and extracranial large artery atherosclerosis (54.9%).^27^ In contrast, studies from the West seem to have a much lower percentage of high hsCRP levels. Den Hertog et al.^20^ and Dewan et al.^28^ noted that 15% and 14.9% of patients with large artery atherosclerosis, respectively, had high CRP levels. After adjustment using multiple logistic regression, high hsCRP was an independent predictor in this group (odds ratio: 2.1, 95% CI: 1.3-3.8) and these findings were advocated by Ladenvall et al.^29^

### Cardioembolic stroke

We found 83.3% of our cardioembolic patients had high hsCRP levels. The other study from India by Rajeshwar et al. also reported an increased prevalence of high hsCRP levels in 58.3% of patients with cardioembolic stroke.^27^ Lower prevalence was noted in other regions of the world; 13.8% in Nepalese, and 24% in Dutch populations.^20, 28^ Increased prevalence of high hsCRP levels in cardioembolic stroke seems to be a unique feature of the Indian population. The underlying cardiac disease in the high hsCRP group was varied and included ascending aorta stenosis (4 patients), congestive heart failure (3 patients), mitral stenosis (4 patients), atrial fibrillation (6 patients), and rheumatic heart disease (3 patients). Our study demonstrated that high hsCRP was significantly associated with cardioembolic stroke (Odds ratio: 3.4 95% CI: 1.9-10.5) and is an independent predicator of cardioembolic stroke.

### Small vessel disease

Compared to large artery atherosclerosis and cardioembolic stroke, the prevalence of high hsCRP levels was lower among stroke patients due to small vessel disease. The 50% prevalence found in our study is similar to that detected by others. Muir et al. observed that (21/96) 21.8% of acute ischemic patients with small vessel disease had elevated CRP > 10 mg/L and 29.8% of lacunar patients from Nepal had high CRP levels.^19, 28^ Den Hertog reported that 13% of patients with small vessel disease had high hsCRP.^20^ Rajeshwar et al. observed a 12.6% prevalence of high hsCRP levels.^27^ The association of high hsCRP with different stroke subtypes in different populations may be secondary to unknown interactions with genetic and environmental pathogenetic factors.

### Stroke of other determined etiology

In this study, patients with stroke of other determined etiology had 40% prevalence of high hsCRP levels (3 patients had hyperhomocysteinemia, 2 had deficiency of protein C, and 1 patient antithrombin III deficiency), which is comparable to that noted by Rajeshwar et al. (25.9%).^27^

### Stroke of undetermined etiology

In our study we noted that 49% of patients with stroke of undetermined etiology had high hsCRP levels. These findings were advocated by Rajeshwar et al. (36.7%).^27^ Den Hertog also confirmed these findings and noted 44% with high CRP (> 7 mg/dl) levels.^20^ The literature is meagre on this stroke subtype, and multiple logistic regression showed that high hsCRP is not an independent predictor of stroke of undetermined etiology.^8^

### Atherogenicity of CRP

CRP, an acute-phase protein synthesised by hepatocytes, is released in the blood stream in response to inflammation and tissue damage.^30, 31^ CRP stimulates the endothelial cells to produce various adhesion molecules, such as intracellular adhesion molecule-1, vascular cell adhesion molecule-1, and E-selectin.^32, 33^ These molecules allow migration of mononuclear cells and T lymphocytes into the vessel wall and play a key role in the formation of atherosclerotic plaque.^34, 35^ CRP also helps in releasing of superoxide anion and stimulation of tissue factor activity.^36^ In addition it induces plasminogen activator inhibitor-1 (PAI-1); a marker of disrupted fibrinolysis and atherothrombosis.^37–39^ Finally, CRP may increase the chance of endothelial cell lysis, and plaque erosion, and can precipitate acute ischemic stroke or coronary syndrome. All these, thus, predispose to atherosclerosis in cerebral and cardiac circulation.

### Association between gender and hsCRP

In our study, the proportion of men with high hsCRP levels was significantly higher (95; 73%) compared to low hsCRP (38; 47.5%); and recent studies have found similar findings.^41^ Devaraj et al.^42^ and Wakugawa et al.^9^ found that raised hsCRP level was an independent risk factor for future ischemic stroke only in men and not in women. Endogenous estrogen has been shown to protect the development of atherosclerosis, and has an anti-inflammatory effect in women.^43, 44^ However, Muir et al. did not find any association between gender and elevated CRP (> 10 mg/L) levels in acute ischemic stroke patients.^19^ Recent studies have demonstrated a fivefold increase in the risk of any vascular event in women with the highest CRP levels.^7, 23^ Thus, elevated CRP level may cause more damage in women.

### Association between age and hsCRP

We demonstrated that high plasma hsCRP level was significantly associated with older age in our patients; similar to the British population.^19^ Rost et al. found elevated CRP level to be a significant predictor of future risk of ischemic cerebrovascular accident in the elderly.^45^ Large prospective studies in apparently healthy subjects have confirmed the prognostic relevance of CRP in the elderly.^46, 47^

### Mortality in the hospital

In the present study, 9 (4.2%) patients died in the hospital due to the disease progression. The Mortality rate was significantly higher in our patients with high hsCRP. Studies in Nepal, Norway, and China had similar findings.^12, 27, 48^ Furthermore, the impact of CRP on mortality seems to be long-term. A recent study showed that elevated CRP levels in young patients with ischemic stroke were associated with an increased risk of mortality, even 12 years after the CRP measurements.^49^

## Conclusion

In conclusion, this study demonstrated that high levels of hsCRP are prevalent in all ischemic stroke subtypes, and are independently associated with large artery atherosclerosis and cardioembolic stroke. In stroke subtypes, high hsCRP levels were associated with a 3-fold increase in risk of developing cardioembolic stroke and a 2-fold raise in risk of large artery atherosclerosis. Hence, in these subtypes high hsCRP may be a marker to initiate primary preventive strategies. In the Jupiter trial, statins decreased the risk of myocardial infarction and ischemic stroke in patients with high hsCRP levels.^50^ Thus, high hsCRP levels may be a marker for starting therapy with statins for both primary and secondary prevention. Future large scale studies are required to explore these findings.
